# Experience‐Dependent Reorganization of Hippocampal CA3 Neuronal Ensembles Associates With Memory Generalization

**DOI:** 10.1002/advs.76661

**Published:** 2026-07-17

**Authors:** Da Song, Zilu Zhu, Yujun Deng, Yujie Song, Hui Li, Junjun Ni, Zhenzhen Quan, Yan Yan, Hong Qing

**Affiliations:** ^1^ Key Laboratory of Molecular Medicine and Biotherapy School of Life Science Beijing Institute of Technology Beijing China; ^2^ Department of Biology Shenzhen MSU‐BIT University Shenzhen Guangdong China; ^3^ The First Hospital of Hebei Medical University Shijiazhuang Hebei China

**Keywords:** hippocampal CA3, memory generalization, neural ensemble dynamics, population decoding

## Abstract

Memory generalization is essential for adaption to novel circumstances through experiential learning, leading to behavioral flexibility and survival capability. However, its underlying neural mechanisms remain to be elucidated. This study designed 8‐arm‐maze‐based tasks to reveal how the hippocampal CA3 associates with the generalization of spatial working memory. Mice successfully transferred the learned rule to novel task configurations, but the efficiency was inversely correlated with task difficulty. In vivo electrophysiological recordings of the CA3 showed that single‐unit and population activity in the CA3 reflected this behavioral transition. On testing day 1, neuronal firing and population trajectories robustly distinguished in relatively simple tasks, but not in more difficult tasks. On testing day 2, as behavioral performance improved, the representational differences of the CA3 neuronal population across different tasks gradually decreased. Decoding analysis revealed that task discriminability based on population activity decreased over time, indicating CA3 neural coding is shifting toward a more generalized pattern. Feature elimination analysis further demonstrated that CA3 neurons employ a sparse but redundant coding scheme to support generalization. Together, the observed neural and behavioral changes are consistent with the emergence of generalized task representations, indicating experience‐dependent reorganization of CA3 population activity during memory generalization across task configurations.

## Graphical abstract

Mice can transfer the learned rule of spatial working memory to guide similar but novel tasks. Hippocampal CA3 populational activity dynamically reorganize during memory generalization, shifting from task‐specific to generalized coding over testing days. Sparse yet redundant neural representations of CA3 enable rule transfer and cognitive map formation, indicating how the brain generalizes for flexible behavior.

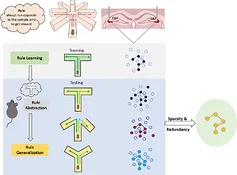

## 1 Introduction

Faced with complicated and volatile environments, both humans and animals possess the ability to generalize prior knowledge from past experiences to adapt to novel circumstances [1, 2]. This generalization ability enables them to identify commonalities and relationships among diverse events, objects, and actions through semantic learning, concept learning, category abstraction, and structure learning, thus applying this ability to various highly intelligent behaviors such as perception, learning, decision‐making, and future planning [3]. The ability to organize knowledge for generalization is widely considered one of the main functions of cognitive mapping, which guides flexible behavior via internal neural representations, accounts for the relationships between events and inferences the consequence of actions [4, 5].

Evidences have shown that cognitive map encompass multiple domains, from psychological concepts of schemas to the mental frameworks for understanding learning sets, etc, but current research on cognitive maps primarily focuses on their neural representation in spatial cognition, especially after the discovery that hippocampal place cells encode spatial navigation cues [6]. Hippocampal place cells exhibit maximal firing when a specific spatial place field is occupied, and this firing is not responding to simple sensory stimulus or specific motor behaviors [7, 8]. The hippocampus not only encodes spatial information, but also maps non‐spatial dimensions. Hippocampal neurons have also been reported to response to visual [9], auditory [10], and olfactory cues [11, 12] or combinations of those. Under multisensory environments, hippocampal place cells can reorganize their population representations in response to the changing factors of environmental geometric [13] or nongeometric cues (odor, color, etc) [14, 15]. When the hippocampus encodes both non‐spatial and spatial features of experience, it introduces a complex and highly organized ensemble to build a schematic framework for multiple related memory elements [16]. The Tolman‐Eichenbaum Machine experiments demonstrate that hippocampal place cells may preserve and transfer structural knowledge through cross‐ environments remapping [17], especially in spatial and rational memory tasks. Therefore, accumulated evidence suggests that the hippocampus plays a crucial role in generalization.

In studies on humans and primates, Garvert MM team showed the research on participants’ choice tasks that these tasks are adaptively influenced by spatial predictive cognitive maps constructed in the hippocampus. These cognitive maps are used for reward generalization [1], suggesting that hippocampal cognitive maps play a role in the updated flexibility of inference. A study on primates also found that hippocampal neurons encode location in abstract value space. Individual neurons in the primate hippocampus construct a map for abstracting cognitive variables through representations of similar location [18]. In macaque, schema cells in the hippocampus were displayed to be responsible for generalization across environments via extracting generic spatial or task schemas from repeated experiences [19]. In a study on rodents, Lin LN's team reported that when a mouse perceives nests or a bed, hippocampal CA1 neurons selectively fire or cease firing, suggesting that hippocampal neurons can extract basic features and commonalities from various episodic experiences and then generalize them into abstract concepts and knowledge from behavioral experiences [20]. Sun C et al., found a specific type of neuron, called event‐specific rate remapping (ESR) cells, was discovered in the CA1 region of the hippocampus. These cells encode generalized information from a designed task rather than precise sensory information. This suggests that activity patterns in the CA1 region not only reflect events but also generalize these events as rule‐based experiences [21]. It has been reported that specific place cell populations in the CA1 region can present orientation and topology of the environment. This orientation selectivity is due to the redistribution of place cells remapping, indicating that prior experience generalization improves the predictability of future environmental representations [22]. Given the heterogeneity of hippocampus subregions in encoding experiences in new environments between CA1 and CA3 [23], it remains unclear whether CA3 contributes to the generalization of spatial working memory rules across task configurations. We have previously demonstrated the important role of hippocampal CA3 in the retrieval of spatial working memory [24], However, whether CA3 activity is associated with the transfer of learned rules to novel but structurally similar tasks remains to be determined.

To examine the neural representation of hippocampal CA3 in memory generalization, we developed a novel behavioral paradigm in an 8‐armed maze experiment that mice completed a series of tasks with the same abstract structure but different physical layouts. Behavioral tests showed that mice were able to learn and transfer the rule from an initial T‐maze task to similar but novel tasks. Notably, the efficiency was correlated with task difficulty, which was defined by the angle difference between the sample and the choice arms in the maze. Through single‐unit recording and neural trajectory decoding, we found that the neural activity of CA3 neurons dynamically reconfigured in accordance with the transferring of tasks. During transferring across tasks, the sparsity of the CA3 neuron population decreased, and the overlap of representations increased, indicating a shift in their encoding pattern toward a more general direction, thus supporting the transfer of common rules. In conclusion, these findings reveal an association between behavioral improvement across tasks and dynamic reorganization of CA3 population activity, with neural representations becoming more convergent over time. This pattern is consistent with the interpretation that improvement in task performance is accompanied by a dynamic reorganization of CA3 population activity, moving from task‐specific representations toward more convergent encodings that may reflect shared task structure rather than low‐level sensory features.

## 2 Results

### 2.1 Mice Generalize From Rule of Spatial Working Memory Across Tasks

To evaluate the task‐transferring ability of mice in spatial working memory, mice sequentially performed a set of spatial working memory tasks in a self‐designed automated 8‐arm maze. These tasks had the same structure but employed different physical layouts. The behavioral diagram is consisted of two stages: training and test. The training stage was a learning process in which the mice learned the features of the spatial working memory task. The test stage contained novel but structurally similar tasks that required the mice to apply the previously abstracted feature to guide subsequent events. The maze arms were numbered from 0 (the main arm) to 7 (choice arms) (Figure S1A). During training, mice performed a classic T‐maze task, eg, the “026” task comprises of the main arm (0), a sample arm (2), and a choice arm (6). Training lasted five days, with the number of trials increased from 20 to 60 per day. Each trial consisted of three phases: sample, delay, and choice (see Methods and previous work [24]). Taken the task 026 as an example, in the sample phase, the door for arm 2 opened to allow the mouse to enter and get a food reward (sweet milk). When the mouse initiated the sample run with the main arm open, the time point was marked as “sample begin”. The delay phase was defined as 3 s by which the mouse was forced to stay after it returned to the main arm 0; in the choice phase, with the doors of arm 0, 2, and 6 all opened, the mouse had to choose the arm opposite to the sample phase in order to receive the reward, which is considered as a correct choice. If the mouse selected the same arm as that in the sample phase, it is considered as a false choice. The time was marked as “choice begin” when the mouse started the choice run. Mice were trained on two mirrored task variants: 026 and 062 (collectively termed as tasks 26). It can be found that the success rates of mice were gradually increased with the number of training days, exceeding 80% after 7 days of training (Figure S1B), indicating that the mice learned the spatial working memory task rules based on the T‐maze. The test stage lasted 4 days, during which mice needed to perform 60 tasks in total per day. Different tasks were randomly presented each day with similar percentages (Figure 1A). On days 1 and 2, mice performed three task types differing in the angular separation between the two test arms: Tasks 26 (180°), Tasks 17 (270°), and Tasks 35 (90°). Each task type included two mirrored configurations (e.g., tasks 26 contain task 026: main arm 0‐sample arm 2‐choice arm 6; and task 062: main arm 0‐sample arm 6‐choice arm 2). Tasks 26 were familiar from training, while Tasks 17 and 35 were novel. On Days 3 and 4, mice performed gradient tasks where the angular separation between test arms progressively decreased within a session: 026‐025‐024‐023 and 062‐064‐063‐065 (angles decreasing from 180° to 45°).

**FIGURE 1:**
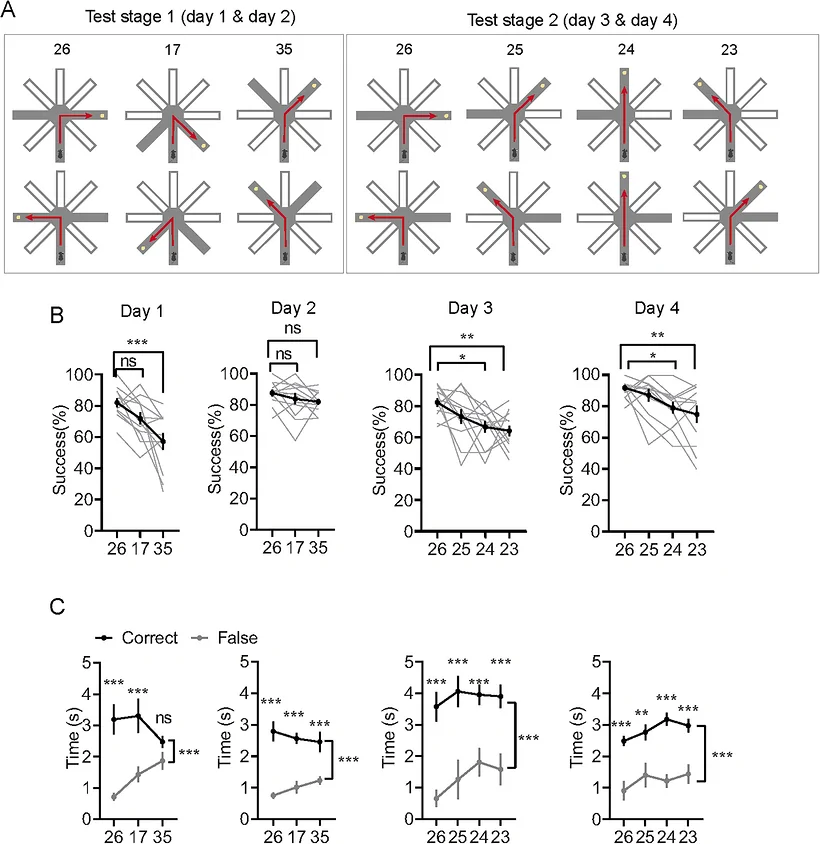
The behavioral performance of WT mice on testing days of task transfer. (A) Schematic diagram of the behavioral paradigm for the test phase of spatial working memory transfer. Red lines in upper and lower panels both indicate the main arm (arm 0) and the correct choice arm (upper: arm 6, 7, 5 on the first and second day of testing and arm 6, 5, 4, 3 on the third and fourth day of testing; lower: arm 2, 1, 3 on the first and second day of testing and arm 2, 3, 4, 5 on the third and fourth day of testing). The yellow dot stands for the reward. (B) Success rates across different task types from test day 1 to day 4 (from left to right) (n = 12, 8 of males and 4 of females). **Left**, One‐way repeated measures ANOVA: *p* = 0.001, F (2, 22) = 9.364. Bonferroni post hoc tests: 26 vs. 17: *p* = 0.187, t = 1.753, df = 22; 26 vs. 35: *p* < 0.001, t = 4.303, df = 22. **Middle left**, One‐way repeated measures ANOVA: *p* = 0.176, F (2, 22) = 1.882. Bonferroni post hoc tests: 26 vs. 17: *p* = 0.441, t = 1.261, df = 22; 26 vs. 35: *p* = 0.139, t = 1.908, df = 22. **Middle right**, One‐way repeated measures ANOVA: *p* = 0.005, F (3, 33) = 5.246. Bonferroni post hoc tests: 26 vs. 25: *p* = 0.238, t = 1.81, df = 33; 26 vs. 24: *p* = 0.011, t = 3.123, df = 33; 26 vs. 23: p = 0.003, t = 3.628, df = 33. **Right**, One‐way repeated measures ANOVA: p = 0.004, F (3, 33) = 5.512. Bonferroni post hoc tests: 26 vs. 25: p = 0.946, t = 1.02, df = 33; 26 vs. 24: *p* = 0.028, t = 2.762, df = 33; 26 vs. 23: *p* = 0.003, t = 3.673, df = 33). (C) Latency from choice initiation to entering the end of the choice arm (for reward or non‐reward) for correct vs. false choices across different task types from test day 1 to day 4 (from left to right). **Left**, Two‐way ANOVA: p < 0.001, F (1, 66) = 33.48. Bonferroni post hoc tests: Task 26: *p* < 0.001, t = 5.009, df = 66; Task 17: *p* < 0.001, t = 3.786, df = 66; Task 35: *p* = 0.672, t = 1.228, df = 66. **Middle Left**, Two‐way ANOVA: *p* < 0.001, F (1, 66) = 78.3. Bonferroni post hoc tests: Task 26: *p* < 0.001, t = 6.498, df = 66; Task 17: *p* < 0.001, t = 4.94, df = 66; Task 35: *p* < 0.001, t = 3.888, df = 66. **Middle right**, Two‐way ANOVA: *p* < 0.001, F (1, 88) = 64.2. Bonferroni post hoc tests: Task 26: *p* < 0.001, t = 4.593, df = 88; Task 25: *p* < 0.001, t = 4.406, df = 88; Task 24: *p* = 0.004, t = 3.378, df = 88; Task 23: *p* = 0.002, t = 3.648, df = 88. **Right**, Two‐way ANOVA: *p* < 0.001, F (1, 88) = 74.59. Bonferroni post hoc tests: Task 26: *p* < 0.001, t = 4.24, df = 88; Task 25: *p* = 0.002, t = 3.664, df = 88; Task 24: *p* < 0.001, t = 5.245, df = 88; Task 23: *p* < 0.001, t = 4.123, df = 88).

On the first day of testing, tasks 26 had the highest success rate at 82.1%, tasks 17 had a comparable success rate at 71.9%, suggesting that mice could readily transfer the learned rule from the familiar tasks 26 to the novel but structurally similar tasks 17. But tasks 35 yielded the lowest rate at 57.2%, suggesting the performance gradient inversely correlated with task difficulty, defined by the angular interval between choice arms (Figure 1B, left). On testing day 2, the success rates for tasks 17 and 35 improved to 91.9% and 83.8%, respectively (Figure 1B, middle left). This improvement across tasks indicates that the mice progressively refined their ability to apply the learned rule feature to novel configurations. We also statistically analyzed the choice latency, defined as the time from the opening of the main arm door to entry into the test arm endpoint. Across all tasks, the latency for correct choices was consistently longer than that for false choices. Furthermore, as task difficulty increased, the latency for false choices gradually increased (Figure 1C, left, and middle left). On the third day and fourth day of testing, the behavioral patterns were similar to those observed on the first day and second day of testing. Success rates gradually declined as angular separation decreased in a single test, but overall performance improved from the third day to the fourth day of testing (Figure 1B, middle right and right). Similarly, the latency for correct choice was longer than that for false choices, and increased with task difficulty (Figure 1C, middle right and right). These results indicated that mice could transfer the learned rule to novel but relatively simple task configurations. For more challenging tasks, however, the transfer efficacy was progressively improved with additional experience. Notably, progressive learning of individual layouts may also contribute to this improvement.

### 2.2 Hippocampal CA3 Exhibits Dynamic Experience‐Dependent Reorganization to Support Memory Generalization

To explore the firing dynamics of hippocampal CA3 neurons during memory generalization, a 64‐channel single‐unit recording was performed to record the firing activity of individual neurons in the bilateral CA3 region of mice across tasks, especially on the first day and the second day of the testing (Figure 2A). On the first day of testing, there were 679 well‐isolated neurons (including 346 neurons from the left CA3 hemisphere and 333 neurons from the right) recorded from wild‐type (WT, n = 7) mice. Of these, 68.78% of the neurons were activated and responsive to different phases of tasks. Based on the activation time, those responsive neurons were categorized into three groups, “prediction firing” neurons (preNs) that fire before mice initiated the choice phase (176 out of 679, 25.92%); “main arm firing” neurons (maNs) that fire when mice started running to the main arm (200 out of 679, 29.46%); and the “choice arm firing” neurons (caNs) that fire when mice run from choice arm to the choice reward (91 out of 679, 13.4%) (Figure 2B).

**FIGURE 2:**
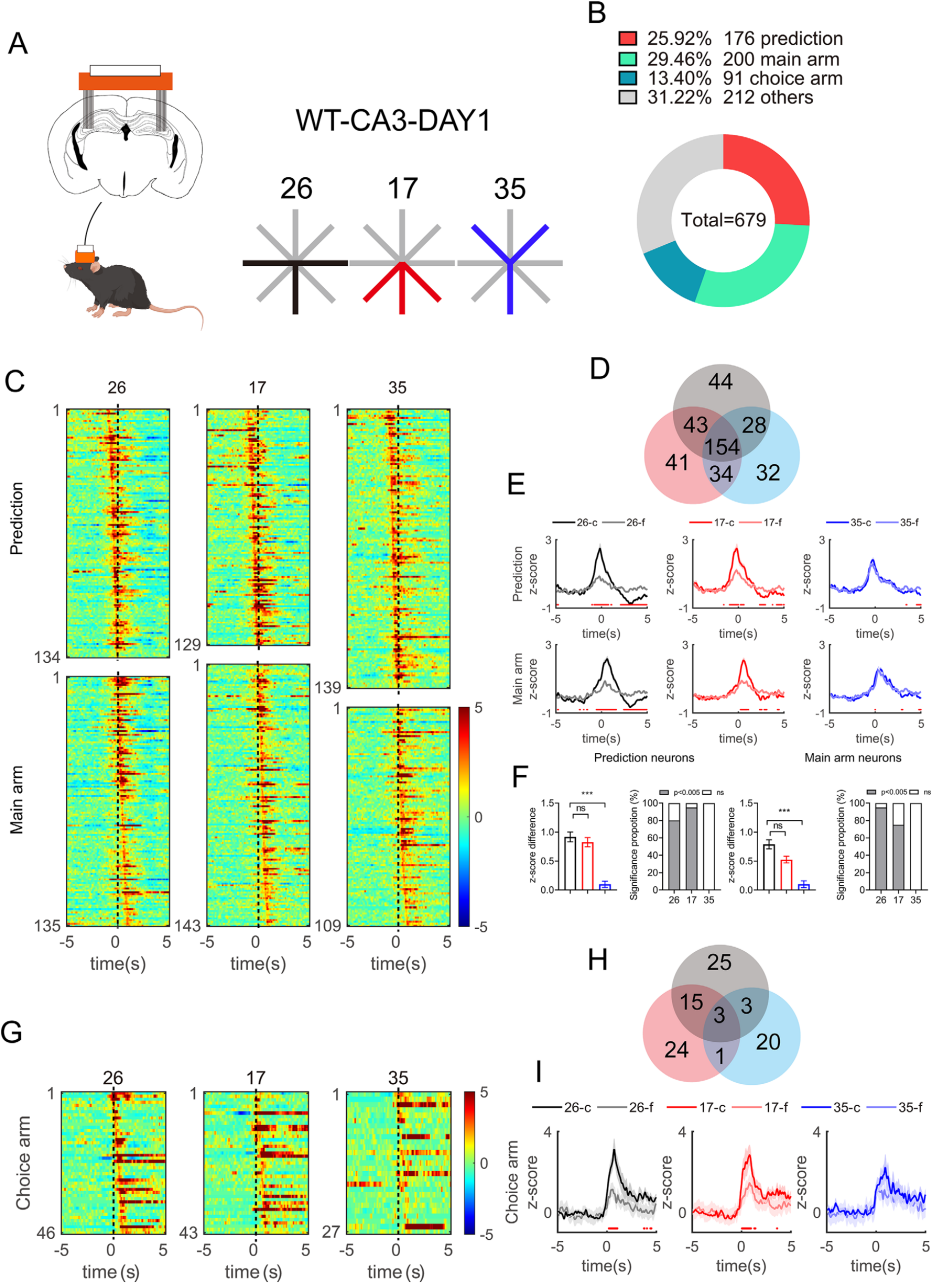
Single‐neuron firing in hippocampal CA3 on test day 1 of task transfer. (A) Schematic illustration of 64‐channel electrode implantation in mice and the spatial working memory behavioral paradigm. Tasks 26 were marked in red color, tasks 17 in red color, and tasks 35 in blue color. (B) Proportional distribution of 679 CA3 neurons recorded during the choice phase, categorized by response type (n = 7 male). Neurons activated during the choice phase were classified into: prediction neurons, main‐arm neurons, and choice‐arm neurons. (C) Neural activity changes (within a ±5‐second window around choice onset) for prediction neurons and main‐arm neurons across three different task types (Tasks 26, 17, 35). Heatmaps display the average firing rate (converted to z‐scores) of individual neurons for each task type within these time points. Color intensity represents the magnitude of the z‐score. (D) Distribution of prediction and main‐arm neurons across the three task types. A total of 154 neurons showed elevated activity in all three task types; 44, 41, and 32 neurons showed elevated activity exclusively in Tasks 26, 17, and 35, respectively; and 43, 28, and 34 neurons showed elevated activity in two of the three task types. (E) Average z‐scores (mean ± SEM) for prediction and main‐arm neurons during correct (dark color) versus false (light color) trials across the three task types. Red data points near the horizontal axis indicate time points with a statistically significant difference *(p < 0.005*) in firing activity between correct and false trials. (F) the difference in average z‐scores (Left) and the proportion of time with statistically significant differences (Middle right) between correct and false trials for prediction and main‐arm neurons during the (−1, 0) and (0, 1) second epochs. (Left, Kruskal–Walli's test: *p* < 0.001; Dunn's multiple comparisons: 26 vs. 17: p >0.999, 26 vs. 35: *p* < 0.001. Middle right, Kruskal–Walli's test: *p* < 0.001; Dunn's multiple comparisons: 26 vs. 17: *p* >0.999, 26 vs. 35: *p* < 0.001). (G) Neural activity changes (within a ±5‐second window around choice onset) for choice‐arm neurons across the three different task types. Heatmaps display the average firing rate (converted to z‐scores) of individual neurons for each task type. Color intensity represents the magnitude of the z‐score. (H) Distribution of choice‐arm neurons across the three task types. Three neurons showed elevated activity in all three task types; 25, 3, and 3 neurons showed elevated activity in two tasks; and 25, 25, and 20 neurons showed elevated activity in only one task type (tasks 26, 17, and 35, respectively). (I) Average z‐scores (mean ± SEM) for choice‐arm neurons during correct (dark color) versus false (light color) trials across the three task types. Data are presented as mean ± SEM; ns, not significant; ^*^ *p* < 0.05, ^**^ *p* < 0.01, ^***^ *p* < 0.005.

We also statistically analyzed the proportion of active neurons in three types of tasks. The proportions of preNs and maNs remained similar across three types of tasks, though individual neurons showed selectivity for either one type, or two types, or three types of tasks (Figure 2C,D). Compared to tasks 26 and 17, tasks 35 engaged a greater number of preNs but fewer maNs. Furthermore, the firing rates of both preNs and maNs were significantly higher for correct than for false choices at the time points of 1s before and after the choice begin of tasks 26 and 17, but no such difference was observed in tasks 35 (Figure 2E). Z‐scores analyses showed more pronounced activity differences between correct and false choices in tasks 26 and 17 than tasks 35 (Figure 2F). These data suggest that preNs and maNs in CA3 differentiate between successful from unsuccessful performance in relatively simple tasks (like tasks 26, and 17), but not in the most difficult tasks 35. For caNs, we further classified these neurons based on their responses to different test arms, especially in correct choices. A certain percentage of firing neurons that are responsive to the choice arm 2(6) also showed firing in response to the choice arm 3(5) or the choice arm 1 (7) and 3 (5). The overlap of caN response profiles was greater between tasks 26 and 17 than that between tasks 26 and 35 (Figure 2H). Furthermore, the z‐score changes of caNs were more significant in tasks 26 and 17 than tasks 35 (Figure 2I). These results indicate that neurons encoding choice arms in familiar tasks are partially “remapped” to encode arms in novel tasks, especially in similar tasks 17, where more neurons are remapped.

In order to intuitively observe the firing pattern of neurons that are responsive to the choice phase, we visualized the firing patterns of typical caNs as an example in relation to temporal and spatial information. Some caNs were selectively activated only in one task type, either in tasks 26, 17, or 35 (Figure S3). Meanwhile, some caNs exhibit higher firing rates in two or more arms in different types of tasks (Figure 3). For example, some caNs show increased firing rates in response to tasks 26 and 17 (Figure 3B). There also caNs with firing higher rates at similar locations in tasks 26 and 35 (Figure 3C), and caNs with higher firing rates in response to tasks 26, 17, and 35 (Figure 3A,D–F). These results demonstrate that a subset of caNs encode analogous spatial information (choice arm reward location) across different task configurations.

**FIGURE 3:**
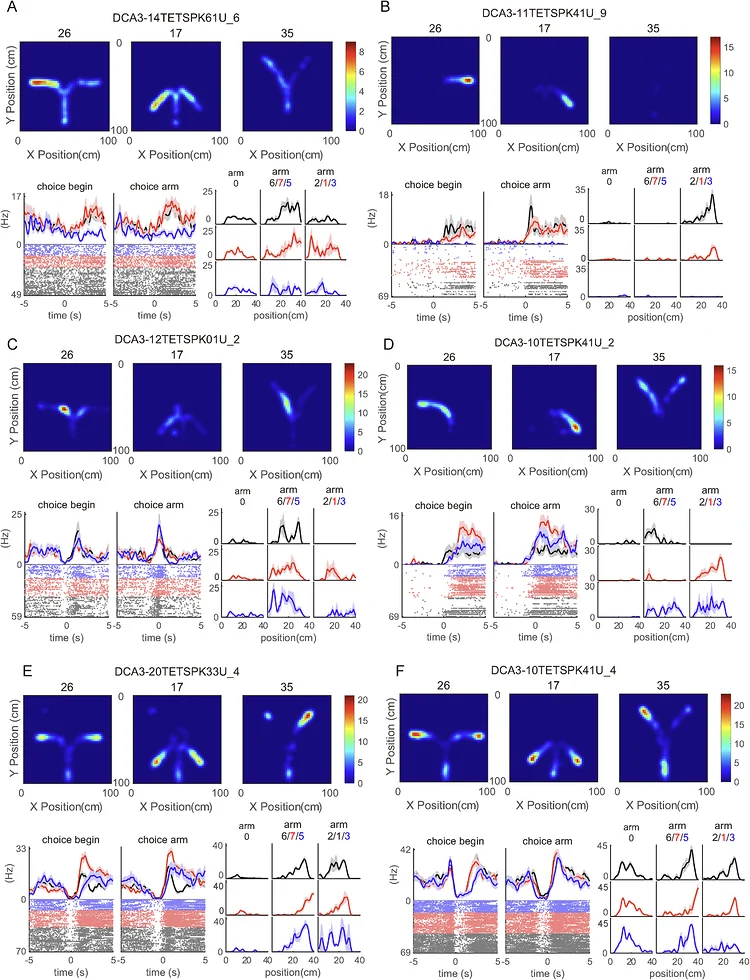
Neurons with specific firing properties in different task types during task transfer. (A,B) Neurons exhibiting firing activity in both tasks 26 and 17. (C) A neuron exhibiting firing activity in both tasks 26 and 35. (D–F) Neurons exhibiting firing activity on multiple arms across tasks 26, 17, and 35. For each neuron: the **top panel** displays a 100 cm × 100 cm firing rate map (50 × 50 resolution) constructed based on the neuron's activity. The **bottom‐left panel** shows the peri‐event raster plot and peri‐event histogram aligned to the choice initiation time point and the choice‐arm entry time point. The **bottom‐right panel** presents a one‐dimensional linear firing rate histogram across different maze arms. The black curve represents the firing activity of neurons in task 26; the red curve for tasks 17 and the blue curve for tasks 35.

On the second day of testing, a total of 631 neurons were recorded (66.4% responded), with proportions of 161 preNs (25.52%), 184 maNs (29.16%), and 74 caNs (11.73%) (Figure S2A). Analysis of the number distribution of preNs and maNs in response to different types of tasks revealed that 199 neurons responded to all three tasks, 86 neurons for two tasks, and only 60 neurons responded to one task (Figure S2B,C). In addition, there is no significant differences in neuronal firing between correct choices and false choices across the three types of tasks (Figure S2D,E). The firing pattern of caNs was similar to that on the first day of testing (Figure S2F,G). These data suggest that neuronal convergence across tasks is consistent with behavioral changes. This is consistent with the behavioral test, where mice exhibited a higher success rate in task 35 on the second day of testing than on the first day. These data implicate CA3 neuronal ensembles dynamically reorganize to represent task‐difficulty‐dependent activities and support the transfer of learned rules to novel tasks.

### 2.3 Dynamic CA3 Population Trajectories can Track Task Difficulty and Behavioral Performance

To further determine the populational changes of CA3 neurons in different tasks, we utilized principal component analysis (PCA) to project populational activity in a low‐dimensional firing‐rate space and trace plot neural trajectories for the entire neural representation during key epochs: 1s before (−1,0) and after (0,1) the choice begin and 1s after entering the choice arm (0,1). On the first day of testing, the overall patterns of neural trajectories for three types of tasks were similar (Figure 4A). However, the Euclidean distances between the neural trajectories for tasks 17 vs. 26 and tasks 35 vs. 26 increased significantly during the epoch of entering the choice arm (Figure 4B). Statistical analysis further showed significant pairwise differences among all tasks across the three time points (Figure 4C, the values in the bar chart exceeded the dashed line). We further quantitated the lengths of neural trajectory for each task and found that, tasks 26 showed different neural trajectory lengths compared to tasks 17 at different time epochs, especially at 1s after choice begin and choice arm, but showed much longer trajectory lengths than tasks 35 (Figure 4D). When the neural trajectory was plotted based on correct and false trials (Figure 4E), we found that the Euclidean distances between the correct and false choices was largest in tasks 26, decreased in tasks 17, but barely unchanged in tasks 35 (Figure 4F,G). This indicates that the neuronal population in the CA3 region could discriminate between correct and false choices in tasks 26 and tasks 17 but not in tasks 35. Additionally, the trajectory lengths for correct choices were significantly longer than for false choices in tasks 26 and 17, but this difference in trajectory lengths between correct and false choices was not statistically significant in tasks 35 (Figure 4H). This further indicates that the rate of change in firing frequency among neuronal populations in tasks 35 varied far less between correct and false trials compared to tasks 26 and 17.

**FIGURE 4:**
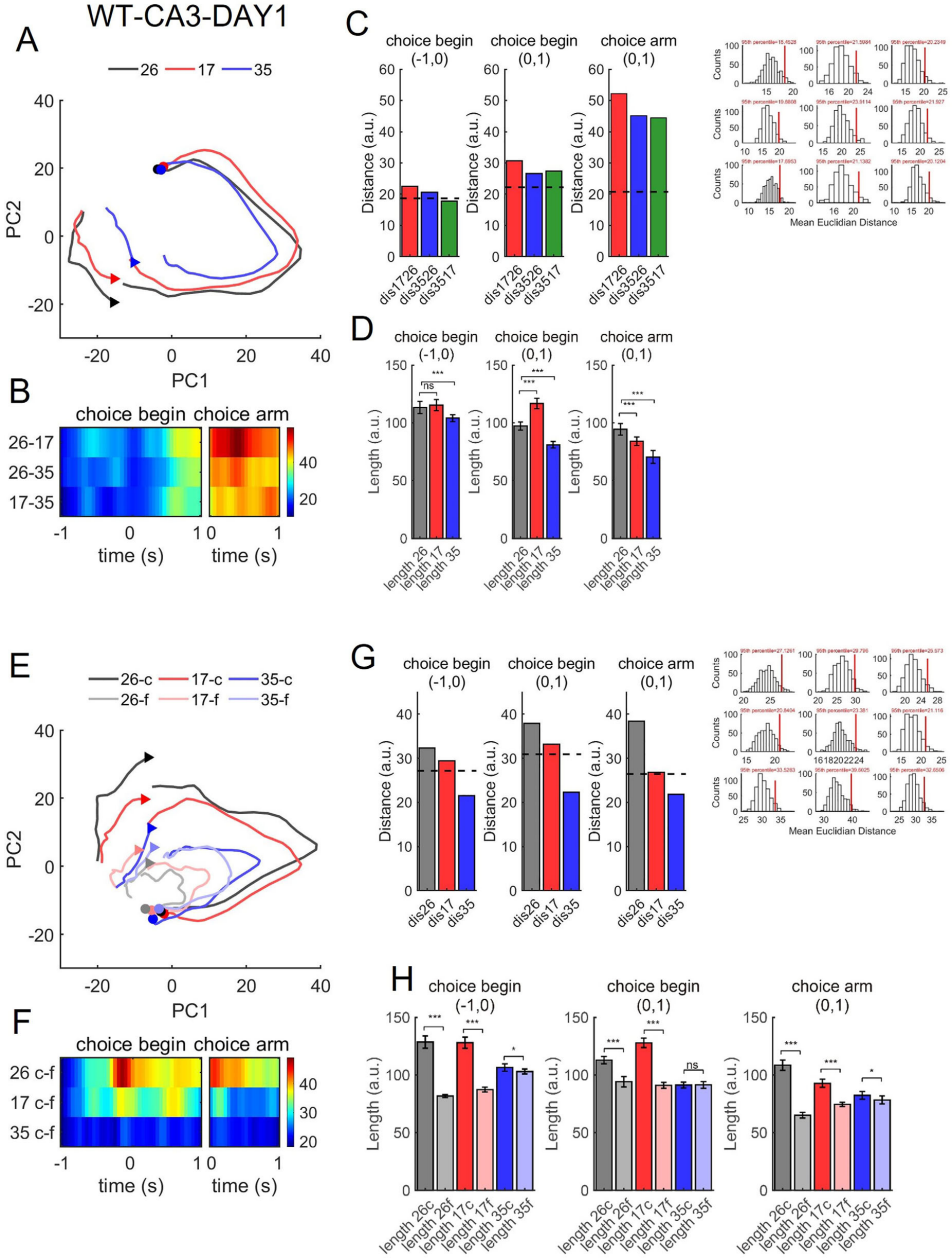
Neural trajectory analysis of CA3 neurons across task types on testing day 1 of task transfer. (A) Neural trajectories after PCA dimensionality reduction for three task types, shown for the time points of 1s before and after choice initiation, and for the 1s after choice‐arm entry. (B) Heatmaps depicting the pairwise Euclidean distances between neural trajectories of the three task types at different time points. (C) The average Euclidean distance for each task type during the three time points: 1s before choice initiation, 1s after choice initiation, and 1s after choice‐arm entry. The dashed line represents the 95% of the average Euclidean distance from randomized data; values exceeding this threshold indicate a significant difference between two neural trajectories. The inset in the top right corner shows the distribution of Euclidean distances from 500 iterations of randomized data, with the red vertical line marking the 95% of the average Euclidean distance from randomized data. The mean of this distribution corresponds to the dashed line value in the main plot. (D) Trajectory lengths for the three task types across the three specified time points. (E) Neural trajectories (after PCA) for correct versus false trials within each task type, shown for the 1s before and after choice initiation and the 1s after choice‐arm entry. (F) Heatmaps of the Euclidean distances between neural trajectories of correct and false trials for each task type at different time points. (G) The average Euclidean distance between correct and false trial trajectories for each task type during the three time points. (H) Trajectory lengths for correct versus false trials in the three task types across the three specified time points.

On the second day of testing, the neural trajectories for three types of tasks were similar to the pattern on the first day of testing, with significant pairwise distances among trajectories, especially at the time epoch of 1s after choice arm (Figure S4A–C). There were no differences between the trajectory length of tasks 35 and those of tasks 26 or 17 at the time epoch of 1s after choice arm on test day 2 (Figure S4D). When the trials were separated based on correct and false choices, the trajectory distances between correct and false trials were similar across the three task types, but significant changes were observed at 1s after the choice‐arm time epoch (Figure S4E–G). Moreover, the trajectory lengths for correct choices were consistently longer than those for false choices across all three task types (Figure S4H). Among these, tasks 35 exhibited remarkable changes from test day 1 to day 2, developing a more significant difference between correct and false trial trajectories, consistent with behavioral improvement of tasks 35 from day 1 to day 2 of testing.

### 2.4 SVM Decoding Shows a Shift From Task‐Specific to Generalized Population Coding of CA3 During Memory Generalization

To investigate how CA3 neurons encode across different tasks, we further employed a support vector machine (SVM) decoder to classify trials based on population activity. Decoding accuracy was assessed across the three time epochs. The data showed that on both the first and second days of testing, the decoding accuracy gradually increased after the choice begin time epoch and peaked at the choice arm time epoch (Figure 5A,C). The peak decoding accuracy on test day 1 was higher than that on test day 2 (Figure 5B). Similarly, the average decoding accuracy at 1s after the choice arm time epoch on test day 1 was higher than that on test day 2 (Figure 5D). This observation indicates a correlation between improving behavioral performance and a gradual reduction in the representational dissimilarity of CA3 population activity across different task conditions. To calculate the decoding accuracy matrix and visualize the test results of different classifiers trained at different time epochs, including the training time (t1) at whole time dimension (−1, 1s) and other test time (t2), we plotted the cross‐temporal decoding matrices of the whole time period and presented them as heatmaps (Figure 5E,F). The CA3 neurons exhibited dynamic coding characteristics, as evidenced by high decoding accuracy on the diagonal. Similar patterns were observed on both the first and second days of testing during memory generalization (Figure 5E,F). Further statistical analysis of the proportion of cross‐temporal decoding matrix entries exceeding 90% of the average decoding accuracy within the ±1s before and after the choice‐arm time epochs showed that this proportion was significantly lower on the testing day 2 compared to the testing day 1 (Figure 5G), suggesting that the encoding selectivity of CA3 neurons on the second day of testing was much higher than that on the first day of testing at the intra‐task time scale.

**FIGURE 5:**
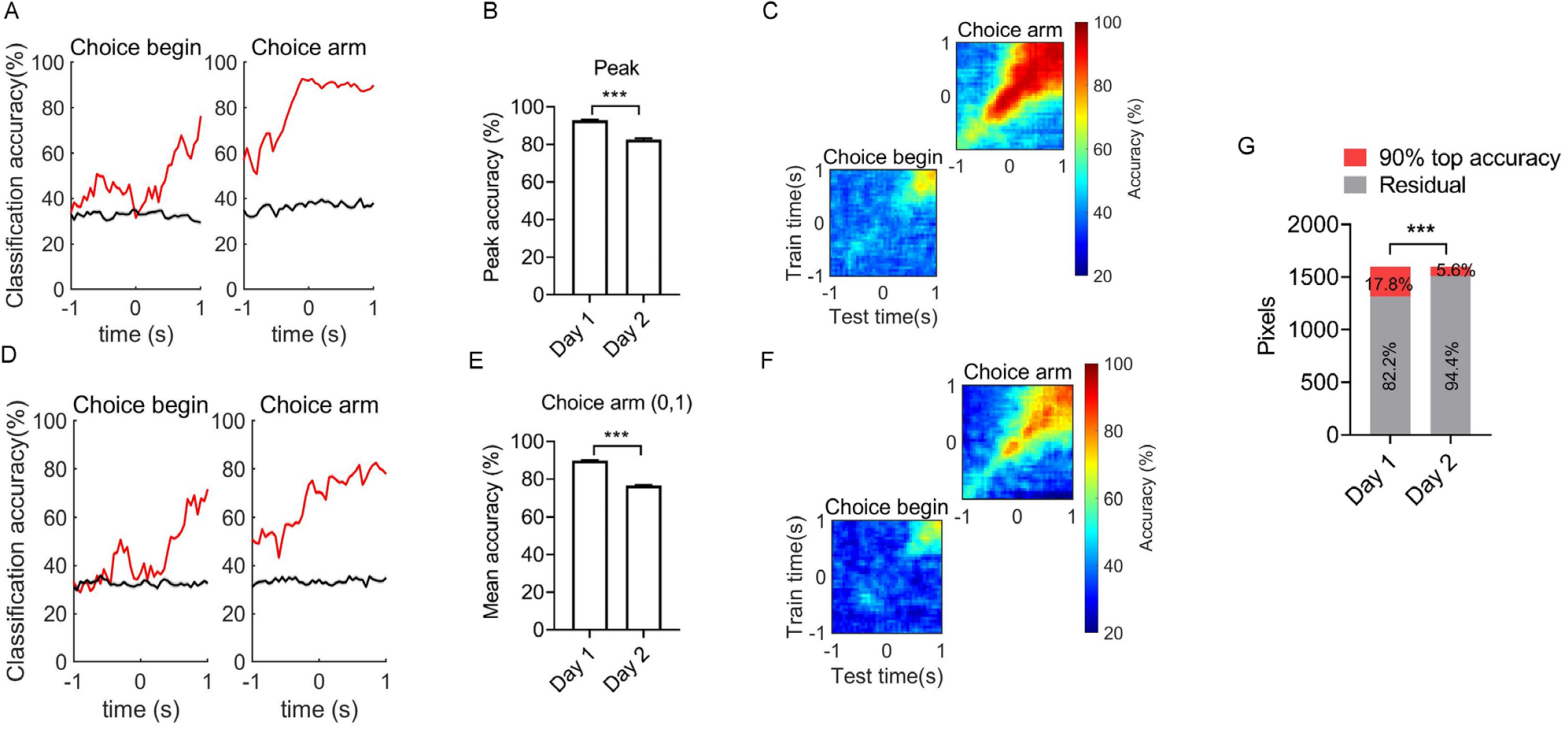
SVM decoding of CA3 population neurons on testing day 1 and day 2 of task transfer. (A,D) Decoding accuracy for classifying the three task types by CA3 neuronal populations, shown for the time points of 1s before and after the choice initiation and choice‐arm entry on testing day 1 (A) and day 2 (D), respectively. (B) Bar graph comparing the peak decoding accuracy between testing day 1 and day 2 (Mann‐Whitney test, *p* < 0.001). (C,F) Cross‐temporal decoding heatmaps for testing day 1 (C) and day 2 (F), respectively. The heatmaps show the decoding accuracy when classifiers trained at time points on the *Y*‐axis are tested on data from time points on the *X*‐axis (within the ±1‐second windows around choice initiation and choice‐arm entry). Color intensity represents the decoding success rate at each (train time, test time) coordinate pair. (E) Bar graph comparing the mean decoding accuracy during the 1s after choice‐arm entry between testing day 1 and day 2 (Mann–Whitney test, *p* < 0.001). **G**: Distribution of pixels (from the heatmaps in C and F) with decoding accuracy exceeding 90% of the peak accuracy (Fisher's test, *p* < 0.001). Data are presented as mean ± SEM; ns, not significant; ^*^p < 0.05, ^**^p < 0.01, ^***^p < 0.005.[Correction added on 24 July 2026, after first online publication: Figure 5 has been updated in this version.]

To further analyze the decoding performance during the time epoch of 1s after the choice arm, we employed the Support Vector Machine‐recursive feature elimination (SVM‐RFE) method. After initial model training at each time epoch, we calculated a criterion score for each neuron's eigenvalue, representing the firing activity of each neuron, and used this score as the basis for feature elimination. In each iteration, we sequentially removed the neuron with the smallest eigenvalue until all features were eliminated. Then, starting from the last eliminated feature, we reversely ordered the accuracy obtained in each iteration. The results revealed that a small subset of CA3 neurons (testing day 1: 93 neurons; testing day 2: 132 neurons) was sufficient to achieve 90% of the maximum decoding accuracy obtained using all neurons (Figure 6A,E for testing day 1; C, G for testing day 2). This suggests that the neuronal ensembles in the CA3 region exhibit a degree of encoding sparsity across memory generalization tasks. Prior to SVM decoding, Principal Component Analysis (PCA) was applied to all neuronal features, and the number of principal components (PCs) was incrementally increased starting from the highest‐ranking components. The results showed that the first 4 PCs (Figure 6B,F) on testing day 1 and the first 17 PCs on testing day 2 (Figure 6D,H) could achieve 90% of the maximum decoding accuracy. When neurons with the highest criterion scores (top‐ranking neurons) were sequentially removed from the feature set of the decoding classifier, eliminating the top 93 eigenvalues on the first day of testing (Figure S5A,E) or the top 132 eigenvalues on the second day of testing still retained 85% of the original decoding accuracy (Figure S5C,G). Likewise, removing the first 4 PCs (testing day 1) or 17 PCs (testing day 2) still retained 88% of the original accuracy on testing day 1 (Figure S5B,F) and 80% on testing day 2 (Figure S5D,H), respectively. This data suggest that CA3 neuronal encoding exhibits a certain degree of redundancy for three types of tasks transferring. We also found that a higher percentage of total neurons (20.9% vs. 13.7%) and PCs (20.9% vs. 5.4%) was required to maintain >90% accuracy on testing day 2 compared to testing day 1 (Figure 6I,J). However, the percentages required after eliminating top neuronal features were similar across two testing days (Figure S5I,J). These findings suggest that achieving behavioral generalization involves broader and less specific neural representations than the initial and more distinct encoding seen on testing day 1, suggesting a shift from high‐fidelity task‐specific encoding to more generalized population encoding.

**FIGURE 6:**
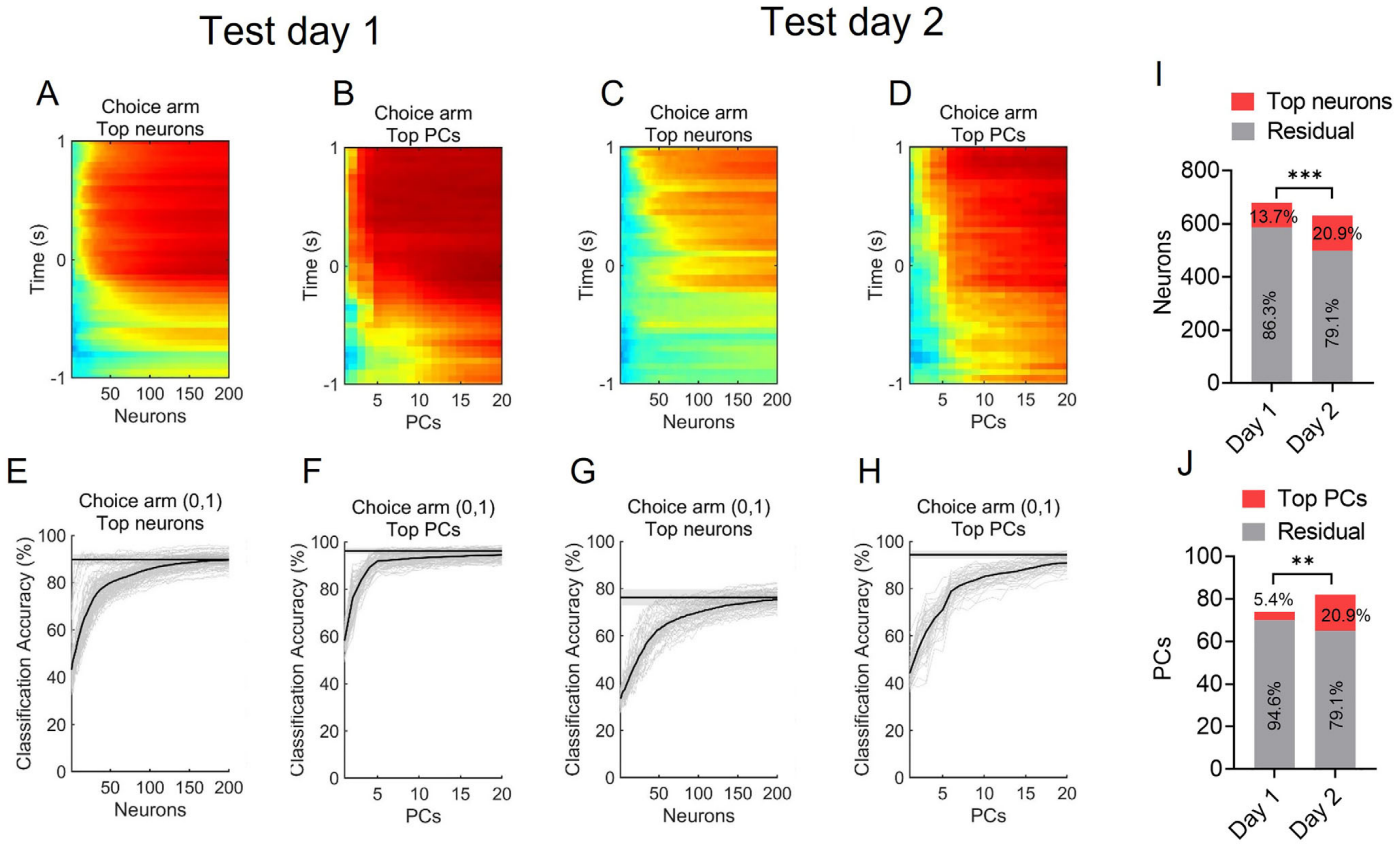
SVM decoding accuracy of CA3 on testing day 1 and day 2 of task transfer. (A) **(testing day 1),** (C) **(testing day 2)**: Heatmaps showing the decoding accuracy achieved by sequentially increasing the number of CA3 neuronal features (sorted by feature importance score from high to low using SVM‐RFE) for the ±1‐second window around the choice‐arm time point. (E) **(testing day 1),** (G) **(testing day 2)**: curves depicting the average classification accuracy as the number of neuronal features is sequentially increased, specifically for the time point of 1s after choice‐arm entry. Light gray lines represent individual decoding iterations. The horizontal line indicates the decoding accuracy achieved using all neuronal features. The shaded area represents the 95% confidence interval (n = 100 iterations). (B) **(testing day 1),** (D) **(testing day 2)**: Heatmaps showing the decoding accuracy achieved by sequentially increasing the number of principal components (PCs, after PCA dimensionality reduction of CA3 neuronal firing data) for the time point of ±1s before and after choice‐arm entry. (F) **(testing day 1),** (H) **(testing day 2)**: curves depicting the average classification accuracy as the number of principal components is sequentially increased, specifically for the 1s after choice‐arm entry. Light gray lines represent individual decoding iterations. The horizontal line indicates the decoding accuracy achieved using the subset of PCs that explains >90% of the variance in the data. The shaded area represents the 95% confidence interval (n = 100 iterations).

## 3 Conclusions

Generalization is a fundamental cognitive process that enables behavior to be flexible and adaptive. In our study, we examined the ability of mice to transfer a learned spatial working memory rule (e.g., ‘choose the arm that was not entered during the sample phase’) to novel but structurally similar 8‐arm maze configurations. Behaviorally, we found that mice readily transferred the “opposite‐arm” rule from the trained T‐maze (tasks 26, 180° separation) to novel but similar layouts. However, immediate generalization performance was inversely correlated to task difficulty, with tasks 35 (90° separation) being the most challenging. This initial performance gradient may stem from increased spatial proximity and potential ambiguity between choice arms, making the abstract rule harder to apply. The significant improvement in performance on tasks 35 on the second day of testing indicates that successful generalization is itself a learning process; with minimal experience, mice refined their ability to apply the learned rule even to the most challenging configuration.

Miranda et al., directly examined remapping of CA3 place cells in a contextual‐ memory retrieval task, which may be contextual‐based generalization [25]. They found that when rats successfully retrieved objects from‐context memory (low object exploration), CA3 showed high spatial correlation (low remapping) between training and testing, consistent with pattern completion. Their pharmacological manipulations further demonstrate that enhancing CA3 NMDAR activity biases the system toward pattern completion (retrieval), whereas enhanced DG NMDAR activity biases toward pattern separation (discrimination). While, in our rule‐learning task, generalization is adaptive: mice extract abstract rules and apply them to similar layouts, which is a form of cognitive flexibility rather than a failure to discriminate. At the neural level, we found CA3 representations become less sensitive to specific layouts and more convergent across tasks. This pattern is consistent with models of schema formation and abstraction in which repeated experience with variable exemplars leads to compressed, generalizable neural codes [26]. However, we acknowledge that alternative explanations remain plausible, including progressive learning of individual task layouts, reduced encoding of novelty, maze habituation, or increased encoding of reward expectations.

The transition to successful generalization by the second day was marked by significant changes in this neural code. Although single‐neuron categorization remained, the differences in firing rates between correct and false trials diminished across the board. Critically, even for tasks 35, population trajectories now showed significant separation between correct and false trials, and the pairwise distances between task representations decreased. Decoding analysis provided converging evidence that the ability of the SVM classifier to distinguish the three types of tasks based on neural activity was lower on the second day than on the first day of testing. This decline in decoding accuracy, couplet with the increased in the number of neurons and principal components required to maintain high classification performance, indicates a “convergence” of population activity patterns across different tasks. This convergence may reflect the extraction and application of general, abstract task structures that reduce the unique neural signatures of each specific configuration while enhancing the shared features that are critical to the rule.

Nevertheless, reduced decoding accuracy across tasks may theoretically reflect a trivial increase in representational overlap (e.g., neurons becoming less selective, noisier, or more broadly tuned) rather than a genuine shift toward rule‐based encoding. In addition, decreases in decoding accuracy may result from multiple reasons, including decreased signal‐to‐noise ratio (SNR), increased trial‐to‐trial variability, or nonlinear representational warping‐ none of which necessarily imply abstraction of universal rules. However, we have carefully reexamined our data and would like to discuss the relevance of these possible factors to reduced decoding accuracy: (1) If reduced decoding accuracy is due to decreased SNR or increased variability, behavioral performance may decrease or become more variable; instead, it improves especially for the hardest task. (2) Nonlinear warping is possible, but it should be highly selective in compressing the distance between‐tasks while preserving or even enhancing separability within‐task (correct vs. incorrect), which is consistent with what we observed. Global nonlinear transformation does not produce this dissociation. While abstraction remains one of several possible explanations, the convergence of behavioral improvement and selective compression of between‐task distances favors this interpretation over trivial alternatives.

Our feature elimination analyses revealed two complementary coding properties in CA3 in this process: sparsity and redundancy. A small subset of neurons was sufficient for high‐accuracy task discrimination, indicating sparse coding. Simultaneously, the system exhibited significant redundancy, as information‐rich neurons or principal components were eliminated, retaining considerable decoding accuracy. This combination may provide a strong and flexible substrate for generalization. Sparse, selective ensembles could anchor core representations, while redundant, distributed activity allows for plasticity and remapping without catastrophic interference, thereby facilitating the transfer of learned structures to new situations. The observed neural dynamics align with theoretical frameworks postulating a role for the hippocampus in relational memory and schema formation [27]. CA3 and its extensive recurrent collaterals are well suited to forming generalized codes by detecting commonalities across related experiences [28].

We also acknowledge that our design does not provide direct causal evidence to completely rule out whether reduced task discriminability and improved behavior are based on generalized coding. A limitation of the current study is that we did not record detailed trajectory or door sampling behavior during the testing stage. Therefore, we cannot exclude the possibility that mice may solve the task through local exploration (i.e., physically checking which doors were open) rather than by generalizing learned rules. This alternative strategy may also explain why latencies increased despite high success rates: successful trials may have involved more exploratory door checks, whereas failures may reflect immediate entry into the sample arm without verifying the choice doors. Future experiments with automated tracking or video analysis are required to help understand whether local exploration could be one of the possibilities leading to correct choices or rule generalization is the main cause for improved behavioral performance.

In addition, there might be other influential factors, such as reduced encoding of novelty, maze habituation, reduced contextual differentiation, and/or increased encoding of reward expectations. However, several features of our data could explain our conclusion that rule generalization is the crucial mechanism for behavioral improvement. First, if reduced novelty encoding or habituation was the primary driver, then performance should improve uniformly across all task difficulties. Instead, the most difficult layout (90°, tasks 35) showed the largest behavioral gains from the first day to the second day of testing, while the familiar layout (180°, tasks 26) remained at ceiling‐ a pattern contrary to habituation. Second, if the animals were simply learning each layout individually, one would expect to acquire layouts within a session learning curve, yet tasks 35 performance on the first day of testing remained stable across trials (without significant improvement) but improved only after sleep, a sign that consolidation depends on abstraction rather than online acquisition of distinct stimulus‐response mappings. Third, our data showed reduced decoding accuracy between tasks on the second day of testing compared to the first day of testing, contradicting the “individual learning” hypothesis, which would predict increasingly distinct neural representations. Although increased reward expectation might be present, it is used as a common signal across tasks, and it does not explain why representations of different task layouts become less discriminable. Finally, it can be seen that mice showed immediate above‐chance performance upon first exposure to tasks 35 (about 60% of success rate). This above‐chance performance argues against a purely layout‐specific learning account and is consistent with the spontaneous application of a previously acquired task rule.

Although our findings are compatible with the interpretation that CA3 population activity converges as animals improve in applying a learned rule, we acknowledge that alternative accounts remain viable. For relatively simple task transitions (e.g., tasks 26 to 17), rule generalization may be achieved with relative ease. For more challenging tasks (e.g., task 35), however, progressive learning of individual maze layouts may also facilitate transfer performance and cannot be discounted. Future work incorporating representational similarity analysis, cross‐task decoding (e.g., training on one task configuration and testing on another), and systematic manipulation of task rules should be considered to clarify the role CA3 activity in abstracting rules or reflecting lower‐dimensional task features.

In conclusion, our study demonstrates that behavioral improvement across tasks is accompanied by progressive reorganization of CA3 population activity, with neural representations becoming more convergent over time. This pattern is consistent with the view that generalization involves an active learning process that reshapes representations beyond simple retrieval of static memory traces, enabling flexible cognitive navigation in changing environments. However, the current evidence supports the presence of experience‐dependent reorganization within CA3 population activity more strongly than it supports the conclusion that the observed neural convergence constitutes a neural representation of an abstracted rule. Future studies directly testing cross‐task generalization and rule manipulation will be necessary to distinguish between these possibilities.

## 4 Materials and Methods

### 4.1 Approvals

All surgical and experimental procedures were approved by the Institutional Animal Care and Use Committee of the Beijing Institute of Technology, Beijing, China (Approval No.: BIT‐EC‐SCXK(京)2019‐0010‐M‐2021113).

### 4.2 Subjects

Adult (2‐4 months old) male wild‐type (WT) (WT, C57/BL6) mice were used and group‐housed on a 12/12‐h light/dark cycle (2‐5 animals per cage) at a consistent ambient temperature (23°C ± 1°C) and humidity (50% ± 5%) with ad libitum food and water. Littermates were randomly assigned to each condition by the experimenter. Male mice were used in most behavioral tests besides the sexual difference experiments. The experiments were performed at day time, and the mice were allowed to rest at night.

### 4.3 Stereotaxic Injections

The animals were deeply anesthetized and placed in a stereotactic frame (RWD, Shenzhen, China). Ophthalmic ointment was applied to prevent dehydration. The virus was injected by a 10 µL Hamilton microsyringe at a constant speed with a microsyringe pump (UMP3; WPI, Sarasota, FL, USA) and controller (Micro4; WPI, Sarasota, FL, USA). For virus injection, AAVretro‐Cre were injected into unilateral CA3 (300 nl), and AAV‐DIO‐Telc‐GFP/ AAV‐DIO‐GFP (300 nl) were injected into contralateral CA3 at the following coordinates: AP: −2.06 mm; ML: ±2.35 mm; DV: −2.35 mm. After viral infection was completed, the needle was held still for 10 min to allow the diffusion of the virus. The needle was then withdrawn slowly and completely.

For in vivo neurophysiological experiments, mice were implanted with a moveable microdriver consisting of a 32 (or 64)‐channel electronic interface board. A total of 8 (or 16) tetrodes were assembled and mounted onto the microdriver. A tetrode consisted of four 25 mm platinum (with 10% iridium) wires (California Fine Wire, USA). The tetrode bundle was then targeted to the CA3 region at the following coordinates: AP: −2.06 mm; ML: ±2.35 mm; DV: −2.35 mm.

### 4.4 Behavioral Task

The spatial working memory generalization test conducted in the eight‐arm maze consists of two main phases: the training phase and the testing phase. In each major phase, mice undergo multiple spatial working memory trials. The key difference between the training and testing phases lies in the choice arms available during testing. We assigned numbers to the different arms of the eight‐arm maze, classifying them as the main arm (labeled arm 0) and test arms (labeled arms 1–7). During the training phase, test arms 2 and 6 were open. In the testing phase, the open choice arms varied depending on the task type.

The training phase typically lasted 7–8 days. The number of trials per day increased gradually from 20 to 60, with increments of 10 trials. Each trial consisted of three phases: the sample phase, the delay phase, and the choice phase, as illustrated in Figure 1. In the sample phase, either arm 2 or arm 6 was randomly opened (here we use opening arm 2 as an example). A food reward (sweetened milk) was provided at the end of arm 2 via a water pump. Simultaneously, the door of arm 0 was opened. The mouse ran from arm 0 to the end of arm 2 to retrieve the reward and then returned to the end of arm 0, at which point the door of arm 0 was closed. During the delay phase, the mouse remained at the end of arm 0 for a 3‐second delay. In the choice phase, the doors of arms 0, 2, and 6 were opened simultaneously. The mouse started from arm 0 and freely choose between arm 2 and arm 6. If the mouse entered the end of arm 2, an infrared sensor was triggered, causing the door of arm 6 to close automatically. No reward was given at the end of arm 2, and the trial was recorded as a false trial. If the mouse entered the end of arm 6, an infrared sensor was triggered, causing the door of arm 2 to close, and a food reward was provided at the end of arm 6. The trial was then recorded as correct. Regardless of the choice, the mouse eventually returned to the end of arm 0, triggering an infrared sensor, after which the doors of arms 0, 2, and 6 all closed, marking the completion of one full trial. A 10‐second inter‐trial interval followed each trial. During the training phase, there were two task types: 26 and 62 (named together as task 26). By 7–8 days of training, the mice typically maintained a success rate above 80%.

The testing phase lasted 4 days, with 60 trials per day. For day 1 and day 2 of testing: six task types were used, including 026, 062, 017, 071, 035, and 053. Task 26 was familiar from the training phase, while tasks 17 and 35 were novel. The grouping was based on the angular configuration of the open arms: task 026 and 062 formed a “T” ‐maze configuration with a 180° separation between arms; task 017 and 071 formed a “个”‐maze configuration with a 270° separation; task 035 and 053 formed a “Y”‐maze configuration with a 90° separation. Thus, tasks were divided into three categories, including two generalization types. For day 3 and day 4 of testing: eight task types were used, including 026, 025, 024, 023, 062, 063, 064, and 065. Tasks 025, 024, 023, 063, 064, and 065 were novel. Tasks were categorized based on the angular separation between the choice arm and the sample arm: 26 and 62 (180°), 25 and 63 (135°), 24 and 64 (90°), and 23 and 65 (45°). The memory generalization ability was analyzed based on the success rates of each test days and between two consecutive days with the same task type.

### 4.5 In Vivo Single‐Unit Recording Analysis

For single‐neuron spike data analysis, we used the MClust 4.4 program (programmed in MATLAB based on A. D. Redish's work, http://redishlab.neuroscience.umn.edu/MClust/MClust.html) to classify single neurons from the action potential data recorded by tetrode electrodes [29, 30, 31].

In MATLAB, after configuring the file paths, we first used the ‘plx2nlx’ script to convert ‘.plx’ files into the ‘.nlx’ file format. Then, the ‘RunClustBatch’ script was used for automatic spike sorting tasks. Subsequently, the ‘MClust’ script was employed for semi‐automatic discrimination and integration of the sorted spike data based on energy, peak‐to‐trough amplitude, and principal component scores. Finally, the firing data for all individual neurons recorded by each tetrode were obtained.

To analyze and classify event‐related neuronal activity in response to tasks, we performed peri‐stimulus time raster plots and peri‐stimulus time histogram (PSTH) analyses for each neuron, centered on specific timestamps. We primarily analyzed three time points during the mouse's choice phase: (1) the moment that the mouse's head passed through the location of the main arm door when it opened (recorded as the choice begin timestamp). (2) The moment that the mouse's head passed through the chosen arm door, i.e., the moment when it just entered the choice arm (recorded as the choice arm timestamp). (3) The moment that the mouse reached the end of the choice arm and received a reward (recorded as the choice reward timestamp). Note: When the mouse made a false choice, it reached the end without receiving a reward. For consistent labeling, the name remains unchanged).

For a specific timestamp ‘t’ from m trials (an m×1 matrix), all action potentials generated by neuron n within a window of 5 s before and after the timestamp were selected. These action potentials were plotted in raster form based on their relative time of occurrence (with the timestamp as zero). The raster plots from all m trials were then aligned and arranged in rows to generate the peri‐stimulus time raster plot. This plot allowed us to observe the firing characteristics of neuron n around the timestamp of interest, containing information from each individual trial.

To further quantitatively analyze changes in the neuron's response strength to the timestamp, the 10s window was divided into 200 bins using 50 ms time points. For each trial and each bin, the number of spikes was counted and divided by 50 ms to obtain the neuron's firing frequency at each moment. The firing frequencies for each time bin were then averaged across all trials to obtain the neuron's time‐varying (over 10 s) firing frequency profile for that timestamp, presented as a histogram—the peri‐stimulus time histogram (PSTH).

Since the absolute firing rates of different neurons could vary significantly, we calculated the z‐score of the firing frequency for normalization to characterize each neuron's response strength to the event and observe activity changes across all recorded neurons. The z‐score was calculated using NeuroExplorer software, where FR is the neuron's firing rate, and ‘Confidence_mean’ is the expected mean of FR, assuming FR follows a Poisson distribution (thus, the standard deviation equals the square root of the expected mean). Both firing rate and z‐score line plots were smoothed using a Gaussian kernel (σ = 150 ms). The shaded areas in the line plots represent the standard error of the mean (SEM).

Based on the analysis of z‐score changes, neurons were classified into three functional categories during the choice phase: (1) Prediction Neurons. For the ‘*choice begin*’ timestamp, neurons were classified as anticipatory if their z‐score exceeded twice the standard deviation for any four consecutive time bins within the (−1, 0) s time epoch. (2) Main Arm Neurons. For the ‘*choice begin*’ timestamp, neurons were classified as main arm neurons if their z‐score exceeded twice the standard deviation for any four consecutive time bins within the (0, 1) s time epoch. (3) Choice Arm Neurons. For the ‘*choice arm*’ timestamp, neurons were classified as choice arm neurons if: a) their z‐score did not exceed twice the standard deviation for any four consecutive time bins within the (−1, 0) s time epoch, and their z‐score exceeded twice the standard deviation for any four consecutive time bins within the (0, 1) s time epoch. These three neuron types were mutually exclusive. The classification order was: first, check for anticipatory neurons; second, check for choice arm neurons; third, check for main arm neurons. All classified neuron types were applicable only for response classification during that day's behavioral task.

### 4.6 Neural Trajectory Analysis

For the generalization tests of spatial working memory on the first and second days, task types were categorized into three groups: the “T”maze tasks (26 and 62), themaze tasks (26 and 62), the “个”maze tasks (26 and 62), themaze tasks (17 and 71), and the “Y”maze tasks (26 and 62), themaze tasks (35 and 53). For each task type, a highmaze tasks (26 and 62), thedimensional neural activity space was constructed: each axis represented the zmaze tasks (26 and 62), thescored firing frequency of a single neuron. The perimaze tasks (26 and 62), thestimulus time histograms (PSTHs) of the n neurons recorded on a given day formed a trajectory within this nmaze tasks (26 and 62), thedimensional space. For each neuron's PSTH, we used random downmaze tasks (26 and 62), thesampling to balance the potential bias caused by the imbalance between the number of correct and false trials.

Subsequently, for each task type, principal component analysis (PCA) was performed on this n‐dimensional space to identify representative features capturing the heterogeneity in neural dynamics [32, 33]. PCA is a common dimensionality reduction tool that maximizes variance in the data [34]. The principal component scores were calculated using the following formula:

Score=Data×Coeff

where Data is the original data matrix (200 × n) for PCA analysis, and Coeff is the matrix of principal component coefficients (n × n), representing the eigenvectors of the sample covariance matrix. Since the first two principal components (PC1 and PC2) are the most prominent features within the eigenvalue spectrum, the neural trajectory for each task was projected onto the two‐dimensional plane defined by PC1 and PC2 [35, 36, 37, 38].

Euclidean Distance: We calculated the Euclidean distance between two trajectories at each time point (bin = 50 ms) within this multi‐dimensional space and visualized the results as heatmaps.

Average Distance: For neural trajectories between different task types, the average distance between trajectories over a 1s time period was calculated.

Trajectory Length (Geodesic Length): For each neural trajectory, the Euclidean distance between adjacent time points was calculated and summed to obtain the length of the trajectory.

For all quantitative calculations of trajectory lengths and inter‐trajectory distances, the number of principal components that explained 95% of the variance was used. For statistical comparative analysis, a leave‐one‐out cross‐validation method was employed for trajectory metric analysis. In each iteration, data from neurons recorded at one site were excluded; thus, the number of iterations equaled the total number of recording sites in the experimental group. Importantly, during each iteration, the same Coeff (the PCA coefficient matrix calculated prior to applying the leave‐one‐out procedure) was applied to each neuron. However, the specific set of neurons included in the calculation differed for each iteration of the leave‐one‐out analysis.

### 4.7 Neural Decoding Analysis

To examine whether the three generalization types could be decoded from population neural activity in the CA3 region during the first and second days of the spatial working memory transferring test, we employed a Support Vector Machine (SVM) with a linear kernel for decoding analysis. For the n neurons recorded from m experimental mice; to align the number of trials across different task types, we used the minimum number of trials M performed for any one generalization type among the m mice as the baseline. For each mouse and each task type, M trial rounds were randomly selected from its data. The neural activity data for all n neurons across all mice for these M trials per task type were then aligned to form a three‐dimensional matrix (tm × (type × M) × n), where tm is the number of time points; (type × M) is the total number of aligned trial rounds; and n is the number of neurons. We performed 50 iterations of random trial selection.

For each time point, using generalization type as the label, we performed SVM classification training and validated the model using K‐fold cross‐validation: the generated data was divided into M parts. In each iteration, the training set consisted of 80% of the data subsets, and the test set consisted of the remaining 20%. We randomly selected the same number of trials for data with different task type labels (Every time, the number of trials for each type label in the training set was aligned with the minimum number of trials all mice performed a given task type, which was 7) and performed fivefold cross‐validation. Finally, the decoding success rate was plotted as a curve over time. As a control, we shuffled all labels randomly 100 times and repeated the same SVM training procedure to test the model's baseline performance.

Similarly, to investigate whether correct or false task performance (regardless of task type) could be decoded from CA3 population neural activity, we defined two labels: correct choice and false choice. The remaining processing steps were the same as described above [39, 40, 41].

### 4.8 Support Vector Machine Recursive Feature Elimination Analysis (SVM‐RFE)

Using the same SVM model described above for decoding analysis, for the multi‐class classification of generalization task types (3 classes), we performed pairwise matching to generate three binary classifiers. For each binary classification task, SVM‐RFE analysis was conducted. This is a sequential backward selection algorithm based on the maximum margin principle of SVM. After training the model on the samples, each neuronal feature is scored and ranked. The feature with the smallest score is removed, and the model is retrained with the remaining features for the next iteration, continuing until only one feature remains. For each time point, each iteration of random trial selection was performed 100 times. The decoding success rate for each iteration at each time point was calculated using K‐fold cross‐validation. If there are “t” time points and “n” neurons (n iterations), a “t × n” two‐dimensional matrix is formed. This matrix is then flipped along the *X*‐axis, starting from the last remaining neuron feature to the full set of features, creating a new 2D matrix. This new matrix is visualized as a heatmap. For average analysis over a specific time period tm, the tm × n matrix is averaged along the columns to plot a curve with the number of neurons on the *X*‐axis and decoding accuracy on the *Y*‐axis (100 curves from 100 random training iterations), along with the average of these 100 curves.

Following the iterative SVM‐RFE analysis described above, neurons are ranked from highest to lowest based on their feature scores. Decoding analysis is first performed using all neuronal features. Then, iteratively, the highest‐scoring neuronal feature is removed, and decoding analysis is repeated with the remaining features. This yields a “t × n” matrix, which is visualized as a heatmap. Average analysis over a specific time period tm can be obtained using the same method as above.

Prior to decoding analysis, all neuronal feature values first underwent PCA for dimensionality reduction. Decoding analysis was initially performed using the principal components (PCs) required to explain over 90% of the data variance. Then, iteratively, the PC with the smallest variance was removed, and decoding analysis was repeated with the remaining PCs until only the PC with the largest variance remained. For each time point, each iteration of random trial selection was performed 100 times, and the decoding success rate for each iteration at each time point was calculated using K‐fold cross‐validation. The data presentation is consistent with the methods above. Similarly, after initial decoding using PCs explaining over 90% variance, the PCs with the largest variance were iteratively removed, and decoding analysis was repeated with the remaining PCs until the PC with the smallest variance remained. The data presentation for this procedure is also consistent with the above description.

To investigate the firing rate of individual neurons in an eight‐arm maze, we calculated the time spent by the neuron at different positions (2 cm × 2 cm bins) in a two‐dimensional map and the number of firing spikes at those positions, thereby obtaining the firing frequency at each location. The resulting firing rate map was then smoothed using Gaussian filtering (smoothing radius: 6 cm). When analyzing the firing rate map during the choice phase, the time period from the choice begin to the choice reward in each trial was extracted, and the analyzing speed was at a range of ≥ 4 cm/s.

### 4.9 Statistics

For behavioral tests, the data are presented as mean ± standard error of the mean (SEM). Statistical analysis was performed using GraphPad Prism 9 and MATLAB. For comparisons involving three or more groups of vertically aligned data, repeated measures one‐way analysis of variance (RM one‐way ANOVA) followed by Bonferroni post hoc multiple comparison tests were used. For multiple‐group bar chart data, two‐way analysis of variance (Two‐way ANOVA) with Bonferroni post hoc multiple comparison tests were applied.

For electrophysiological analyses, the Mann‐Whitney test was used for unpaired comparisons between two groups. For unpaired comparisons across three or more groups, the Kruskal–Walli's test followed by Dunn's multiple comparison test was applied. For contingency tables involving two categories, Fisher's exact test was used, while the Chi‐square test was employed for contingency tables with three or more categories. A *p*‐value of < 0.05 was considered statistically significant.

## Author Contributions

ZZ.Q., D.S., and H.Q. conceived and designed the studies. D.S. and ZL. Z. performed optogenetics and single‐unit recording. YJ. D. and H. L. performed behavioral tests; Y.Y. and YJ. S. performed virus injection and immunohistochemistry. D.S., ZZ.Q., and JJ.N. performed data analysis. ZZ.Q. wrote the paper. All authors contributed to the data analysis and presentation in the paper.

## Conflicts of Interest

The authors declare no conflicts of interest.

## Data and Code Availability

All data reported in this paper will be shared by the lead contact upon request. Any additional information required to reanalyze the data reported in this paper is available from the corresponding authors upon request.

## Supporting information




**Supporting File**: advs76661‐sup‐0001‐SuppMat.docx.
