# Supplementary material for: Experience‐Dependent Reorganization of Hippocampal CA3 Neuronal Ensembles Associates With Memory Generalization
Source: Adv Sci (Weinh). 2026 Jul 17;13(57):e76661. doi: 10.1002/advs.76661 (PMC13379258; doi:10.1002/advs.76661)
Supplement: Supplementary file 1 [file ADVS-13-e76661-s001.docx]

**Supplementary figures and figure legends**

**Supplementary figure 1:** 8-armed maze and behavioral performance of mice in the training stage

**A**, **upper panel**, diagram of the 8-arm maze. The main arm is labelled “0”, the other arms are labelled from “1” to “7”, the door for each arm was shown in black rectangle with orange frames. **Lower panel**, the schematic diagram of the spatial working memory generalization behavioral task (tasks 26 as example) during the training phase of the eight-arm maze. **B**, In the training stage, the success rate of mice gradually increased with the number of days and reached over 80% on the sixth and seventh days of training stage (n=12).

**Supplementary figure 2:** Firing Properties of individual CA3 neurons on the testing day 2 of memory generalization

**A**, Proportion distribution of 631 neurons in CA3 recorded during the choice phase of spatial working memory, classified by responsive type. Neurons activated during the choice phase were categorized as prediction neurons, main arm neurons, or choice arm neurons. **B**, Changes in neuronal activity for prediction neurons and main arm neurons across three different task types (tasks 26, 17, and 35) within a 5s time window of the choice initiation. Heatmaps display the average firing rate of individual neurons during the same task type, converted to z-scores. The color gradient represents the magnitude of z-scores, with warm and cool tones indicating higher and lower values, respectively. **C**, Distribution changes of prediction neurons and main arm neurons across the three different task types. Among them, 199 neurons showed increased neuronal activity across all three task types. Additionally, 13, 19, and 28 neurons exhibited increased activity exclusively in task 26, task 17, or task 35, respectively. Furthermore, 17, 29, and 40 neurons showed increased activity in two of the three task types. **D**, Average z-scores for prediction neurons and main arm neurons during correct (dark color) and false (light color) choices across the three task types. Shaded areas indicate the standard error of the mean (SEM). Red scatter points near the horizontal axis denote time points where statistically significant differences (p < 0.005) exist between firing activity for correct and false choices. **E**, Differences in average z-scores between correct and false choices for prediction neurons and main arm neurons during the (-1, 0) and (0, 1) time epochs. No statistically significant differences were observed in z-score differences across tasks 26, 17, and 35 (E1, Kruskal-Walli’s test: p = 0.09; Dunn’s multiple comparisons: 26 vs. 17: p = 0.063; 26 vs. 35: p = 0.48. E3, Kruskal-Walli’s test: p = 0.4324; Dunn’s multiple comparisons: 26 vs. 17: p > 0.999; 26 vs. 35: p = 0.439). **F**, Changes in neuronal activity for choice arm neurons across three different task types (tasks 26, 17, and 35) within a 5s time window centered on the choice initiation. Heatmaps display the average firing rate of individual neurons during the same task type, converted to z-scores. The color gradient represents the magnitude of z-scores. **G**, Distribution changes of choice arm neurons across the three task types. Among them, 2 neurons showed increased neuronal activity across all three task types. Additionally, 11, 1, and 3 neurons exhibited increased activity in two of the three task types. Furthermore, 12, 25, and 18 neurons showed increased activity in only one of the three task types. **H**, Average z-scores for choice arm neurons during correct (dark color) and false (light color) choices across all three task types. Shaded areas indicate the standard error of the mean (SEM). Data are presented as mean ± SEM. ns, not significant; **p < 0.05, **p < 0.01, ***p < 0.005*.

**Supplementary figure3:** Examples of CA3 single-neuron firing activity across three different task types during the testing day 1 and day 2 of memory generalization

**A, B:** Neurons exhibiting firing activity specifically on arm 2 or arm 6 in tasks 26. **C, D:** Neurons exhibiting firing activity specifically on arm 1 or arm 7 in task 17. **E, F:** Neurons exhibiting firing activity specifically on arm 3 or arm 5 in tasks 35. For each neuron: the top panel displays a 100cm×100cm firing rate map with a 50×50 resolution, generated based on the neuron's activity. The bottom-left panel presents the peri-event raster plot and peri-event histogram aligned to the choice initiation time point and the choice-arm entry time point. The bottom-right panel shows the one-dimensional linear firing rate histogram of the neuron across different maze arms.

**Supplementary figure 4:** Neural trajectory of CA3 neurons across three task types on the testing day 2 of memory generalization

**A,** Neural trajectories after PCA dimensionality reduction for the three task types, shown for the 1-second windows before and after choice initiation, and the 1-second window after choice-arm entry. **B,** Heatmaps depicting the pairwise Euclidean distances between neural trajectories of the three task types at different time points. **C,** The average Euclidean distance for each task type during different time epochs: 1s before and after choice begin, and 1s after choice-arm entry. The dashed line represents the 95% of the average Euclidean distance from randomized data; values exceeding this threshold indicate a significant difference between two neural trajectories. The inset in the top right corner shows the distribution of Euclidean distances from 500 iterations of randomized data, with the red vertical line marking the 95th percentile. Its mean corresponds to the dashed line in the main plot. **D,** Trajectory lengths for the three task types across the three specified time windows. **E,** Neural trajectories (after PCA) for correct versus false trials within each task type, shown for the time epochs of 1s before and after choice begin and the 1s after choice-arm entry. **F,** Heatmaps of the Euclidean distances between neural trajectories of correct and false trials for each task type at different time epochs. **G,** The average Euclidean distance between correct and false trial trajectories for each task type during the different time epochs. The dashed line represents the 95% of the average Euclidean distance from randomized data; values above it indicates a significant trajectory difference. The inset shows the distribution from 500 random data iterations, with the red line marking the 95%. Its mean value corresponds to the dashed line in the main plot. **H,** Trajectory lengths for correct versus false trials in the three task types across the three specified time windows.

**Supplementary figure 5:** SVM decoding accuracy of CA3 excluding top neurons on testing day 1 and day 2 of memory generalization

**A (testing day 1), C (testing day 2):** Heatmaps depicting the decoding accuracy after sequentially removing neurons with the highest feature scores (ranked by SVM-RFE) from CA3 neuronal data in the ±1-second window around the choice-arm time point. **E (testing day 1), G (testing day 2):** Curves showing the average classification accuracy in the 1s time window after choice-arm entry as neurons are sequentially removed. Individual decoding iterations are shown as light gray curves. The horizontal line indicates the decoding accuracy achieved using all neuronal features. The shaded area represents the 95% confidence interval (n = 100 iterations). **B (testing day 1), D (testing day 2):** Heatmaps showing the decoding accuracy after sequentially reducing the number of principal components (PCs) derived from PCA of CA3 neuronal firing data in the ±1-second window around the choice-arm time point, using only the subset of PCs that explain >90% of the variance. **F (testing day 1), H (testing day 2):** Curves depicting the average classification accuracy in the 1-second window after choice-arm entry as PCs are sequentially reduced. Light gray curves represent individual decoding iterations. The horizontal line indicates the decoding accuracy achieved using the PC subset that explains >90% of the variance. The shaded area represents the 95% confidence interval (n = 100 iterations).

|  | Animal_ID | Tasks 26 | | Tasks 17 | | Tasks 35 | |  |  |
| --- | --- | --- | --- | --- | --- | --- | --- | --- | --- |
| Testing  day 1 | 1 | 20 | 16 | 16 | 15 | 14 | 10 |  |  |
|  | 2 | 23 | 18 | 15 | 13 | 16 | 13 |  |  |
|  | 3 | 14 | 14 | 11 | 9 | 16 | 4 |  |  |
|  | 4 | 17 | 13 | 16 | 10 | 17 | 5 |  |  |
|  | 5 | 12 | 11 | 21 | 17 | 17 | 6 |  |  |
|  | 6 | 18 | 16 | 13 | 9 | 29 | 15 |  |  |
|  | 7 | 19 | 17 | 14 | 8 | 14 | 8 |  |  |
|  | 8 | 8 | 5 | 15 | 7 | 17 | 10 |  |  |
|  | 9 | 31 | 23 | 22 | 14 | 18 | 11 |  |  |
|  | 10 | 20 | 17 | 16 | 13 | 14 | 10 |  |  |
|  | 11 | 16 | 13 | 11 | 8 | 14 | 10 |  |  |
|  | 12 | 22 | 17 | 18 | 12 | 22 | 16 |  |  |
| Testing  day 2 | 1 | 18 | 15 | 20 | 16 | 22 | 17 |  |  |
|  | 2 | 21 | 15 | 11 | 9 | 20 | 16 |  |  |
|  | 3 | 22 | 19 | 16 | 14 | 15 | 12 |  |  |
|  | 4 | 23 | 18 | 21 | 12 | 16 | 13 |  |  |
|  | 5 | 18 | 16 | 11 | 10 | 11 | 9 |  |  |
|  | 6 | 11 | 10 | 12 | 12 | 17 | 14 |  |  |
|  | 7 | 15 | 13 | 17 | 16 | 8 | 7 |  |  |
|  | 8 | 18 | 17 | 15 | 11 | 17 | 15 |  |  |
|  | 9 | 31 | 23 | 22 | 14 | 18 | 11 |  |  |
|  | 10 | 14 | 14 | 22 | 20 | 14 | 13 |  |  |
|  | 11 | 15 | 13 | 17 | 12 | 28 | 20 |  |  |
|  | 12 | 18 | 16 | 15 | 13 | 14 | 10 |  |  |
| Testing  day 3 | Animal_ID | Task 026/062 | | Tasks 025/063 | | Task 024/064 | | Task 023/065 | |
|  | 1 | 16 | 12 | 15 | 11 | 15 | 11 | 15 | 7 |
|  | 2 | 18 | 16 | 15 | 12 | 15 | 11 | 18 | 15 |
|  | 3 | 17 | 16 | 18 | 13 | 14 | 6 | 9 | 7 |
|  | 4 | 11 | 10 | 9 | 8 | 10 | 8 | 10 | 7 |
|  | 5 | 16 | 12 | 18 | 17 | 14 | 9 | 16 | 12 |
|  | 6 | 17 | 14 | 12 | 6 | 14 | 7 | 17 | 10 |
|  | 7 | 18 | 16 | 12 | 5 | 13 | 9 | 17 | 12 |
|  | 8 | 15 | 14 | 13 | 9 | 18 | 15 | 14 | 8 |
|  | 9 | 19 | 16 | 16 | 11 | 26 | 13 | 11 | 7 |
|  | 10 | 16 | 11 | 11 | 7 | 19 | 13 | 11 | 7 |
|  | 11 | 12 | 8 | 16 | 12 | 16 | 12 | 16 | 8 |
|  | 12 | 15 | 12 | 16 | 15 | 16 | 12 | 18 | 10 |
| Testing  day 4 | Animal_ID | Task 026/062 | | Tasks 025/063 | | Task 024/064 | | Task 023/065 | |
|  | 1 | 13 | 13 | 18 | 17 | 18 | 15 | 17 | 14 |
|  | 2 | 17 | 16 | 12 | 11 | 14 | 12 | 17 | 16 |
|  | 3 | 15 | 15 | 17 | 16 | 13 | 10 | 15 | 7 |
|  | 4 | 19 | 18 | 16 | 12 | 14 | 9 | 15 | 6 |
|  | 5 | 14 | 13 | 9 | 5 | 12 | 8 | 11 | 9 |
|  | 6 | 15 | 14 | 11 | 10 | 23 | 20 | 11 | 7 |
|  | 7 | 16 | 15 | 14 | 12 | 14 | 11 | 16 | 12 |
|  | 8 | 14 | 12 | 13 | 9 | 11 | 6 | 11 | 6 |
|  | 9 | 18 | 16 | 16 | 15 | 13 | 11 | 13 | 12 |
|  | 10 | 14 | 12 | 16 | 16 | 17 | 17 | 13 | 12 |
|  | 11 | 19 | 15 | 11 | 10 | 13 | 10 | 18 | 16 |
|  | 12 | 19 | 17 | 14 | 14 | 15 | 13 | 18 | 15 |

**Supplementary table 1:** Number of Trials in each task type for each mouse during four days of testing (Relative to Figure 1, mice used in behavioral tests)

|  | Animal_ID | Tasks 26 | | Tasks 17 | | Tasks 35 | |
| --- | --- | --- | --- | --- | --- | --- | --- |
| Testing day 1 | DCA3-9 | 23 | 20 | 12 | 8 | 17 | 13 |
|  | DCA3-10 | 26 | 21 | 29 | 21 | 17 | 11 |
|  | DCA3-11 | 20 | 18 | 31 | 26 | 21 | 16 |
|  | DCA3-12 | 23 | 21 | 22 | 19 | 16 | 10 |
|  | DCA3-14 | 27 | 22 | 13 | 12 | 11 | 8 |
|  | DCA3-17 | 23 | 19 | 9 | 5 | 19 | 18 |
|  | DCA3-20 | 33 | 31 | 23 | 18 | 15 | 6 |
|  |  |  |  |  |  |  |  |
|  |  |  |  |  |  |  |  |
|  |  |  |  |  |  |  |  |
|  | Animal_ID | Tasks 26 | | Tasks 17 | | Tasks 35 | |
| Testing day 2 | DCA3-9 | 17 | 15 | 23 | 20 | 22 | 15 |
|  | DCA3-10 | 12 | 10 | 26 | 19 | 16 | 13 |
|  | DCA3-11 | 21 | 16 | 15 | 14 | 25 | 21 |
|  | DCA3-12 | 20 | 15 | 19 | 18 | 21 | 17 |
|  | DCA3-14 | 9 | 6 | 12 | 10 | 12 | 8 |
|  | DCA3-17 | 11 | 10 | 20 | 16 | 19 | 18 |
|  | DCA3-20 | 22 | 20 | 26 | 22 | 22 | 18 |

**Supplementary table 2:** Number of Trials in each task type for each mouse during the first and second day of testing (Relative to Figure 2-4, mice used in *in vivo* electrophysiological experiments)

| Testing day 1 | Animal_ID | Recorded neurons from Left | Recorded neurons from Right |
| --- | --- | --- | --- |
|  | DCA3-9 | 37 | 25 |
|  | DCA3-10 | 40 | 35 |
|  | DCA3-11 | 61 | 67 |
|  | DCA3-12 | 42 | 37 |
|  | DCA3-14 | 36 | 51 |
|  | DCA3-17 | 37 | 11 |
|  | DCA3-20 | 96 | 104 |
| Testing day 2 | Animal_ID | Recorded neurons from Left | Recorded neurons from Right |
|  | DCA3-9 | 39 | 31 |
|  | DCA3-10 | 37 | 36 |
|  | DCA3-11 | 37 | 40 |
|  | DCA3-12 | 43 | 36 |
|  | DCA3-14 | 43 | 54 |
|  | DCA3-17 | 46 | 28 |
|  | DCA3-20 | 78 | 83 |

**Supplementary table 3:** Number of Neurons recorded from left and right CA3 region in 7 mice during the testing day 1 and day 2 (Relative to Figure 2)
